# Neuroprotective Effects of the Neural-Induced Adipose-Derived Stem Cell Secretome against Rotenone-Induced Mitochondrial and Endoplasmic Reticulum Dysfunction

**DOI:** 10.3390/ijms24065622

**Published:** 2023-03-15

**Authors:** Mahesh Ramalingam, Sujeong Jang, Jinsu Hwang, Boeun Kim, Hyong-Ho Cho, Eungpil Kim, Han-Seong Jeong

**Affiliations:** 1 Department of Physiology, Chonnam National University Medical School, Hwasun 58128, Republic of Korea; sujeong.jjang@gmail.com (S.J.); wlstn0128@naver.com (J.H.); 2 Gwangju Alzheimer’s Disease and Related Dementias (GARD) Cohort Center, Chosun University, Gwangju 61452, Republic of Korea; okwoosan@naver.com; 3 Department of Otolaryngology-Head and Neck Surgery, Chonnam National University Hospital, Chonnam National University Medical School, Gwangju 61469, Republic of Korea; victocho@jnu.ac.kr; 4 Biopharmaceutical Research Center, Jeonnam Bioindustry Foundation, Hwasun 58141, Republic of Korea; keungpil@gmail.com

**Keywords:** Parkinson’s disease, rotenone, endoplasmic reticulum, mitochondria-associated membranes, mitochondrial tethering

## Abstract

Mesenchymal stem cells (MSCs) have therapeutic effects on neurodegenerative diseases (NDDs) known by their secreted molecules, referred to as the “secretome”. The mitochondrial complex I inhibitor, rotenone (ROT), reproduces α-synuclein (α-syn) aggregation seen in Parkinson’s disease (PD). In this present study, we examined the neuroprotective effects of the secretome from neural-induced human adipose tissue-derived stem cells (NI-ADSC-SM) during ROT toxicity in SH-SY5Y cells. Exposure to ROT significantly impaired the mitophagy by increased LRRK2, mitochondrial fission, and endoplasmic reticulum (ER) stress (ERS). ROT also increased the levels of calcium (Ca^2+^), VDAC, and GRP75, and decreased phosphorylated (p)-IP3R Ser1756/total (t)-IP3R1. However, NI-ADSC-SM treatment decreased Ca^2+^ levels along with LRRK2, insoluble ubiquitin, mitochondrial fission by halting p-DRP1 Ser616, ERS by reducing p-PERK Thr981, p-/t-IRE1α, p-SAPK, ATF4, and CHOP. In addition, NI-ADSC-SM restored the mitophagy, mitochondrial fusion, and tethering to the ER. These data suggest that NI-ADSC-SM decreases ROT-induced dysfunction in mitochondria and the ER, which subsequently stabilized tethering in mitochondria-associated membranes in SH-SY5Y cells.

## 1. Introduction

Parkinson’s disease (PD) is complex, and the second most common age-related multifactorial neurodegenerative disorder (NDD) characterized by motor and non-motor symptoms that reduce the quality of life. PD can affect all movement, including walking, physical balance, and speech, and is associated with a reduction of a neurotransmitter in the brain called dopamine (DA). Moreover, inhibition of the mitochondrial electron transport chain (ETC) complex I, leading to the production of reactive oxygen species (ROS), mitochondrial dysfunction, α-synuclein (α-syn) aggregation, and oxidative stress, is associated with the occurrence of PD [1,2]. Leucine-rich repeat kinase 2 (LRRK2; PARK8) can be modified by the overexpression of α-syn and might impair mitophagy [3,4] via the phosphatase and tensin homolog (PTEN)-induced putative kinase 1 (PINK1) and the E3-ubiquitin ligase Parkin (PARK2) [5]. Ubiquitin (Ub) is essential for the recognition of desired cargoes for degradation. PINK1 accumulates on the outer mitochondrial membrane (OMM) of damaged mitochondria by interacting with import receptor subunit translocase of the OMM (TOM complex) and activates parkin-mediated ubiquitination of OMM proteins to degrade the mitochondria [6]. The expression of PINK1 and parkin are functionally linked to mitochondrial fission [7] controlled by the dynamin-related protein 1 (DRP1), which translocates from the cytosol to the OMM. However, mitochondrial fusion is controlled by mitofusin 1 and 2 (MFN1/2) localized to the OMM and optic atrophy 1 (OPA1) located in the inner mitochondrial membrane (IMM) [8].

Abnormally increased mitochondrial fission may induce endoplasmic reticulum (ER) stress (ERS) [2]. α-syn aggregates accumulate inside the ER activating key sensing proteins, protein kinase R-like endoplasmic reticulum kinase (PERK) and inositol-requiring enzyme 1 α (IRE1α) [2], via the dissociation of ER chaperone glucose-regulated protein 78 (GRP78)/binding protein (BiP) [9]. PERK-dependent protein translational modifications on the alpha (α) subunit of eukaryotic initiation factor 2 (eIF2α) lead to the paradoxical increase of pro-apoptotic transcription factors, such as activating transcription factor 4 (ATF4) and C/EBP homologous protein (CHOP) [10]. IRE1α activation leads to apoptosis through stress-activated protein kinase (SAPK; c-Jun N-terminal kinase; JNK) signaling [11]. ER membrane proteins interact with the OMM complex to exchange materials and transmit signals between them to maintain and balance cellular activities [12]. The exchange of calcium (Ca^2+^) between these two organelles [13] is regulated by a molecular tripartite tethering complex containing the inositol 1,4,5-triphosphate receptor (IP3R), glucose-regulated protein 75 (GRP75), and the voltage-dependent anion channel (VDAC) [14]. The interface between the ER and mitochondria for Ca^2+^ fluxes, among other cellular functions, encompasses the microdomain and mitochondria-associated membranes (MAM), and is tightly controlled by additional tethering proteins, such as MFNs. Mitochondrial MFN1 and 2 tethering complexes with MFN2 present in the ER membrane also physically connect ER and mitochondria [8].

Rotenone (ROT), a lipophilic piscicidal compound isolated from the roots of the subtropical plant species of *Lonchocarpus* and *Derris* suppresses the flow of electrons from the iron–sulfur centers in mitochondrial electron transport chain complex I. ROT reproduces PD-like impairments, such as decreased tyrosine hydroxylase, increased phosphorylation and aggregation of α-syn, and imbalanced autophagy degradation, which induces apoptotic death in SH-SY5Y neuroblastoma cells [15,16]. Treatments for PD mainly focus on restoring mitochondrial function and subsequently relieving motor symptoms, such as tremors, bradykinesia, and rigidity. Mesenchymal stem cells (MSCs) have the potential therapeutic capacity to replace dopamine and stimulate brain repair [17]. Bioactive molecules secreted from MSCs, referred to as “the secretome”, include growth factors, cytokines, chemokines, microvesicles, and exosomes known for their improved therapeutic effects [18]. Moreover, adipose tissue-derived stem cells (ADSC) have been reported to be easily harvested and can differentiate into neural cells in the presence of basic fibroblast growth factor (bFGF) and forskolin [19,20,21]. In this present study, we evaluated the neuroprotective effects of the neural-induced ADSC secretome (NI-ADSC-SM) on ROT-induced dysfunction of mitochondria, the endoplasmic reticulum, and their tethering proteins in human SH-SY5Y cells.

## 2. Results

### 2.1. Effects of the NI-ADSC-SM on Intracellular Ca^2+^ Levels after ROT Exposure

**Figure 1:**
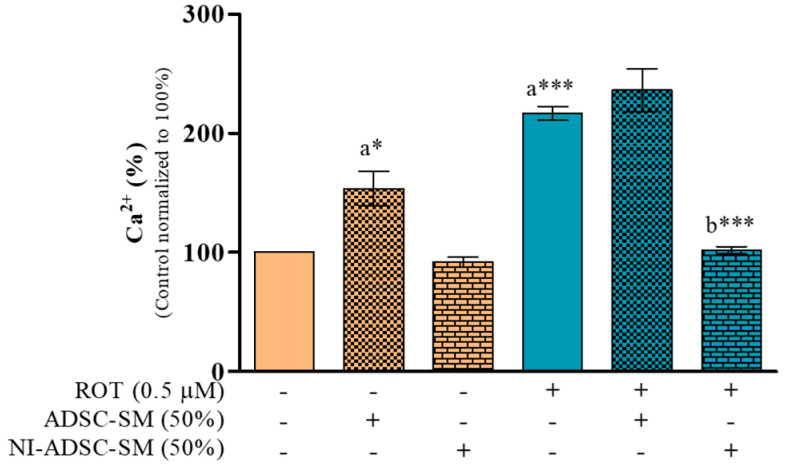
Effect of the NI-ADSC-SM on ROT-induced Ca^2+^ levels. SH-SY5Y cells seeded at 50,000 cells/mL in DMEM containing 1% FBS and incubated overnight were in the presence of ROT (0.5 μM) or DMSO for 48 h alone or with the ADSC-SM or NI-ADSC-SM (each diluted by 50%) during the last 24 h as depicted in Supplementary Figure S1b. Intracellular Ca^2+^ levels were assessed using the fura-2AM assay. Data are shown as the means (bars) and SEM (error bars) of three independent cell culture experiments. Statistical analysis was performed using one-way ANOVA with Tukey’s post-test. Statistical comparisons: a—compared with control; b—compared with ROT; * *p* < 0.05 and *** *p* < 0.001.

In this study, ROT-induced toxicity (48 h) induced higher Ca^2+^ production (Figure 1; 216.6%; *p* < 0.001) compared with control SH-SY5Y cells (100%). The experimental study plan is described in Supplementary Figure S1b. However, treatment with a 50% dilution of the NI-ADSC-SM against ROT toxicity for the last 24 h successively reduced the Ca^2+^ production (101.8%; *p* < 0.001). The ADSC-SM did not reduce the ROT-induced Ca^2+^ levels; however, it showed increased Ca^2+^ levels in control cells. In our previous studies, ADSC-SM treatment significantly decreased the cell survival with increased ROS levels in control cells [15,16]. These results suggest that the NI-ADSC-SM has more protective effects than the ADSC-SM against ROT-induced toxicity in SH-SY5Y cells.

### 2.2. Effects of the NI-ADSC-SM on LRRK2 Protein Expression after ROT Exposure

**Figure 2:**
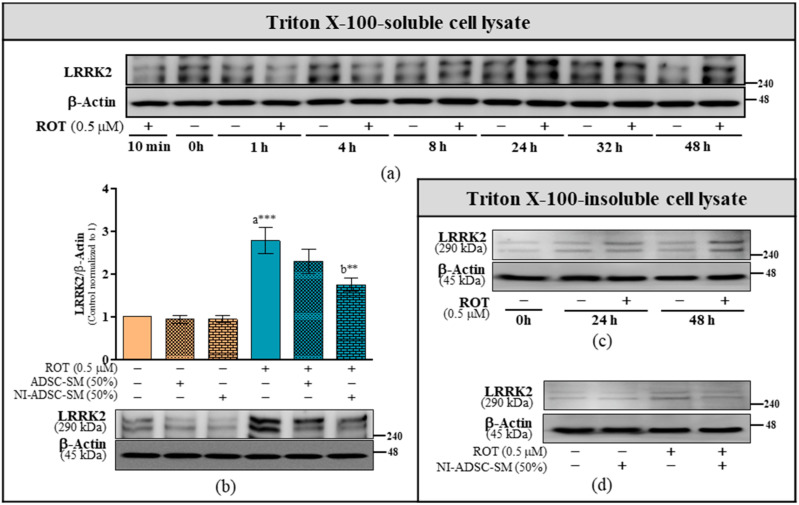
Effects of the NI-ADSC-SM on LRRK2 expression. (**a**,**c**) Cells were treated with or without ROT (0.5 μM) at different timepoints as depicted in Supplementary Figure S1a, and LRRK2 protein expression in Triton X-100-soluble (**a**) and Triton X-100-insoluble (**c**) cell lysate fractions were assessed by Western blotting. (**b**,**d**) Cells were incubated in the presence of ROT (0.5 μM) or DMSO for 48 h alone or with the ADSC-SM or NI-ADSC-SM (each diluted by 50%) during the last 24 h. LRRK2 protein expression in Triton X-100-soluble (**b**) and Triton X-100-insoluble (**d**) cell lysate fractions was assessed by Western blotting. Data are shown as the means (bars) and SEM (error bars) of three independent cell culture experiments. Statistical analysis was performed using one-way ANOVA with Tukey’s post-test. Statistical comparisons: a—compared with control; b—compared with ROT; ** *p* < 0.01 and *** *p* < 0.001.

LRRK2 as one of the most common causes of PD provided much hope for the field of PD therapeutics. As shown in Figure 2, the LRRK2 protein level was significantly increased in SH-SY5Y cells after exposure to ROT for 24 and 48 h in Triton X-100-soluble (a) and -insoluble (c) fractions. At 48 h, ROT significantly increased LRRK2 expression (*p* < 0.001; Figure 2b); however, the levels were decreased by NI-ADSC-SM treatment in the Triton X-100-soluble (*p* < 0.01) and -insoluble fractions (Figure 2b and Figure 2d, respectively). These results suggest that the NI-ADSC-SM prevents LRRK2 expression changes during ROT exposure.

### 2.3. Effects of the NI-ADSC-SM on PINK1 and Parkin Protein Expression after ROT Exposure

**Figure 3:**
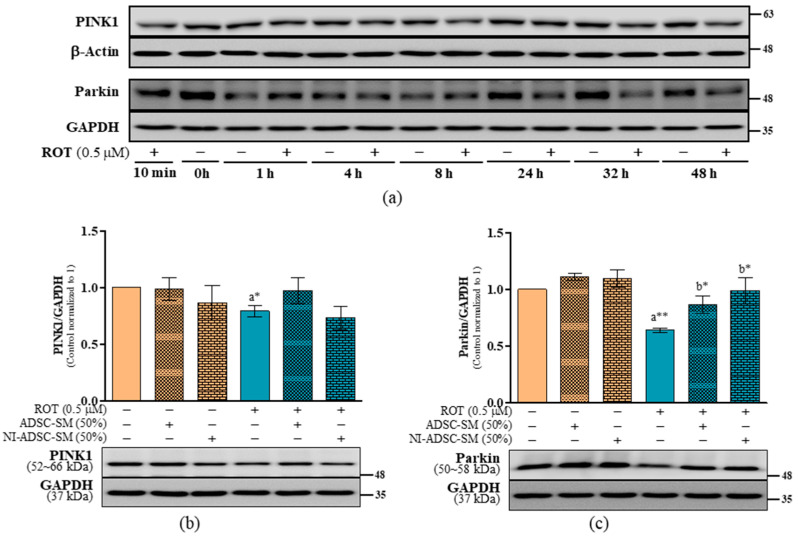
Effects of the NI-ADSC-SM on mitophagy-related proteins. Cells treated with or without ROT (0.5 μM) at different timepoints were assessed for PINK1 and parkin by Western blotting (**a**). Cells were incubated in the presence of ROT (0.5 μM) or DMSO for 48 h and then treated with the ADSC-SM or NI-ADSC-SM (each diluted by 50%) during the last 24 h. The levels of PINK1/GAPDH (**b**) and parkin/GAPDH (**c**) protein expression were assessed by Western blotting. Data are shown as the mean (bars) and SEM (error bars) of three independent cell culture experiments. Statistical analysis was performed using one-way ANOVA with Tukey’s post-test. Statistical comparisons: a—compared with control; b—compared with ROT; * *p* < 0.05 and ** *p* < 0.01.

PINK1 and parkin promote mitochondrial health. In this study, the ROT exposure decreased PINK1 (Figure 3a; Figure 3b: *p* < 0.05) and parkin (Figure 3a; Figure 3c: *p* < 0.01) expression at 48 h in SH-SY5Y cells. Treatment with the NI-ADSC-SM or ADSC-SM increased parkin (*p* < 0.05) levels after ROT exposure (Figure 3c), though they had no effect on PINK1 (Figure 3b). Moreover, neither the ADSC-SM nor NI-ADSC-SM altered PINK1 or parkin expression in the control groups. These results suggest that the NI-ADSC-SM can rescue parkin expression after ROT exposure and may impede dysfunctional mitophagy and the parkin-mediated signaling pathway.

### 2.4. Effects of the NI-ADSC-SM on Ub Protein Expression after ROT Exposure

**Figure 4:**
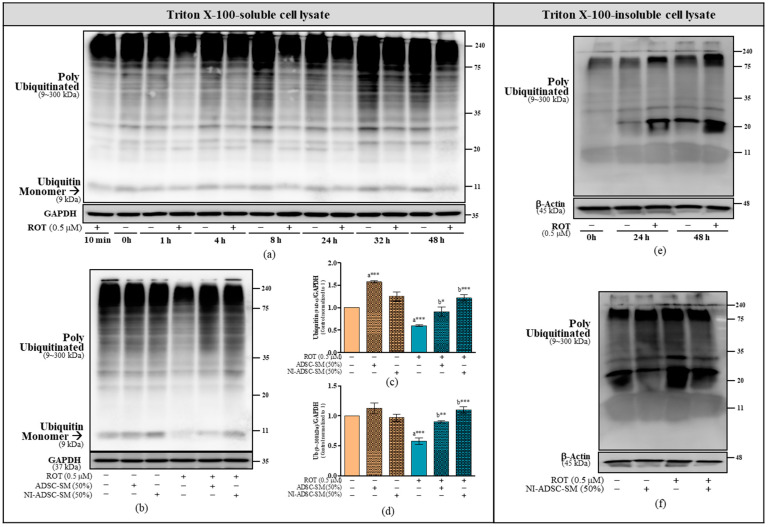
Effects of the NI-ADSC-SM on Ubiquitin (Ub). (**a**,**e**) Cells treated with or without ROT (0.5 μM) at different timepoints were assessed for Ub protein expression in Triton X-100-soluble (**a**) and Triton X-100-insoluble (**e**) cell lysate fractions by Western blotting. (**b**–**d**,**f**) Cells were incubated in the presence of ROT (0.5 μM) or DMSO for 48 h alone or with the ADSC-SM or NI-ADSC-SM (each diluted by 50%) during the last 24 h (**b**). The levels of Ub monomer at 9 kDa (**c**) and poly ubiquitinated at 9~300 kDa in the Triton X-100-soluble fractions (**d**) or in the Triton X-100-insoluble fractions (**f**) were assessed by Western blotting. Data are shown as the means (bars) and SEM (error bars) of three independent cell culture experiments. Statistical analysis was performed using one-way ANOVA with Tukey’s post-test. Statistical comparisons: a—compared with control; b—compared with ROT; * *p* < 0.05, ** *p* < 0.01, and *** *p* < 0.001.

Ub is a substrate for PINK1. Damaged mitochondria are known to be cleared by mitophagy mechanisms by mediating ubiquitination. In our time-course study, ROT decreased the levels of monomer (9 kDa) and polyubiquitinated (9~300 kDa) Ub in the Triton X-100-soluble fraction (Figure 4a), but increased them in the Triton X-100-insoluble fraction (Figure 4e). The levels of monomer (9 kDa; Figure 4b,c) and polyubiquitinated (9~300 kDa; Figure 4b,d) Ub in the Triton X-100-soluble fraction was upregulated by the NI-ADSC-SM (*p* < 0.001) or ADSC-SM (*p* < 0.05 for monomer; *p* < 0.01 for ubiquitinated) treatment after ROT exposure. As shown in Figure 4f, we observed that NI-ADSC-SM decreased the levels of Ub in the Triton X-100-insoluble fraction. The ADSC-SM was not used in Western blot to detect the Triton X-100-insoluble fraction and was shown to be less protective than the NI-ADSC-SM.

### 2.5. Effects of the NI-ADSC-SM on DJ-1 and TOM20 Protein Expression after ROT Exposure

**Figure 5:**
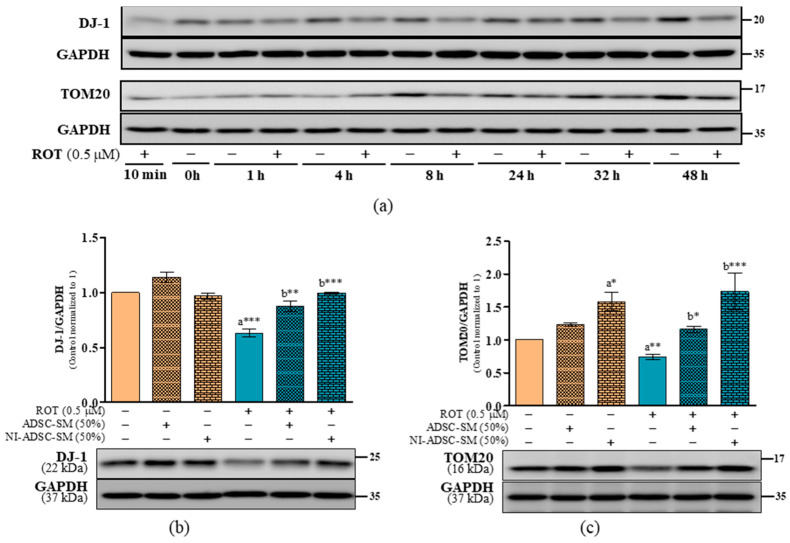
Effects of the NI-ADSC-SM on DJ-1 and TOM20 expression. Cells treated with or without ROT (0.5 μM) at different timepoints were assessed for DJ-1 and TOM20 expression by Western blotting (**a**). Cells were incubated in the presence of ROT (0.5 μM) or DMSO for 48 h alone or with the ADSC-SM or NI-ADSC-SM (each diluted by 50%) during the last 24 h. The levels of DJ-1/GAPDH (**b**) and TOM20/GAPDH (**c**) protein expression were assessed by Western blotting. Data are shown as the means (bars) and SEM (error bars) of three independent cell culture experiments. Statistical analysis was performed using one-way ANOVA with Tukey’s post-test. Statistical comparisons: a—compared with control; b—compared with ROT; * *p* < 0.05, ** *p* < 0.01, and *** *p* < 0.001.

Along with PINK1 and parkin, DJ-1 has various cellular functions. DJ-1 colocalizes with Lewy bodies (LBs) and can downregulate α-syn. Moreover, oligomeric α-syn binds to TOM20, a transit peptide receptor from the OMM, to impair mitochondrial protein import. Using a time-course study, the levels of DJ-1 and TOM20 were significantly decreased by ROT toxicity at 24 and 48 h (Figure 5a). DJ-1 (*p* < 0.001; Figure 5b) and TOM20 (*p* < 0.01; Figure 5c) were significantly decreased after ROT exposure for 48 h. By contrast, the NI-ADSC-SM upregulated both DJ-1 and TOM20 in the ROT-exposed cells (*p* < 0.001). The ADSC-SM also increased DJ-1 (*p* < 0.01) and TOM20 (*p* < 0.05) after ROT exposure.

### 2.6. Effects of the NI-ADSC-SM on Mitochondrial Fission and Fusion Protein Expression after ROT Exposure

**Figure 6:**
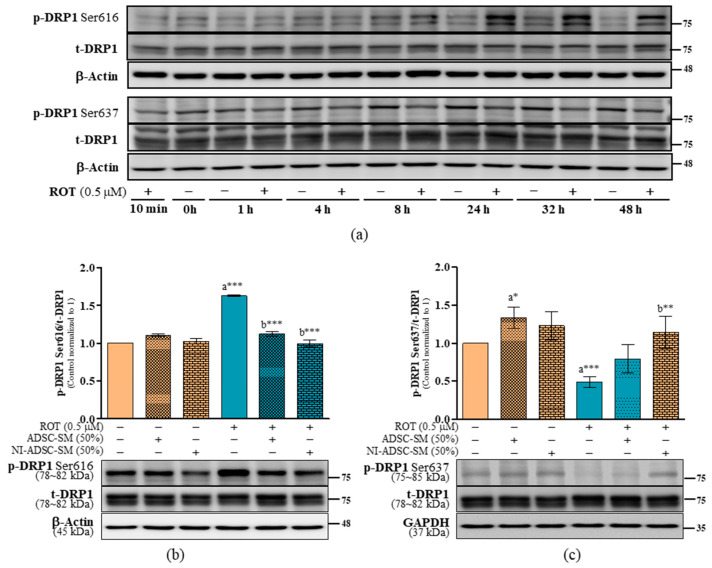
Effects of the NI-ADSC-SM on mitochondrial fission-associated proteins. Cells treated with or without ROT (0.5 μM) at different timepoints were assessed for DPR1 protein expression by Western blotting (**a**). Cells were incubated in the presence of ROT (0.5 μM) or DMSO for 48 h alone or with ADSC-SM or NI-ADSC-SM (each diluted by 50%) during the last 24 h. The levels of p-DRP1 Ser616/t-DRP1 (**b**) and p-DRP1 Ser637/t-DRP1 (**c**) protein expression were assessed by Western blotting. Data are shown as the means (bars) and SEM (error bars) of three independent cell culture experiments. Statistical analysis was performed using one-way ANOVA with Tukey’s post-test. Statistical comparisons: a—compared with control; b—compared with ROT; * *p* < 0.05, ** *p* < 0.01, and *** *p* < 0.001. p-, phosphorylated; t-, total.

Mitochondrial fission is the division of single mitochondria into two and is mainly controlled by DRP1 phosphorylated at Ser616 and Ser637. We found that ROT-induced neurotoxicity significantly increased the phosphorylated DRP1 Ser616 (Figure 6a,b) and decreased the phosphorylated DRP1 Ser637 (Figure 6a,c), although ROT did not modify total DRP1 (t-DRP1) levels (Figure 6). The ratios of p-DRP1 Ser616/t-DRP1 (*p* < 0.001; Figure 6b), p-DRP1 Ser616/β-actin (*p* < 0.05; Supplementary Figure S2a), p-DRP1 Ser637/t-DRP1 (*p* < 0.001; Figure 6c), and p-DRP1 Ser637/GAPDH (*p* < 0.001; Supplementary Figure S2c) were all significant. These results suggest that ROT induced the translocation of DRP1 Ser616 from the cytosol to mitochondria, possibly leading to a malfunction in mitochondrial dynamics. However, the ratio of p-DRP1 Ser616/t-DRP1 was decreased (*p* < 0.001; Figure 6b), and p-DRP1 Ser637/t-DRP1 was increased (*p* < 0.01; Figure 6c) by NI-ADSC-SM treatment. The t-DRP1 was also not changed (*p* > 0.05; Supplementary Figure S2b,d) by the NI-ADSC-SM in SH-SY5Y cells after exposure to ROT.

**Figure 7:**
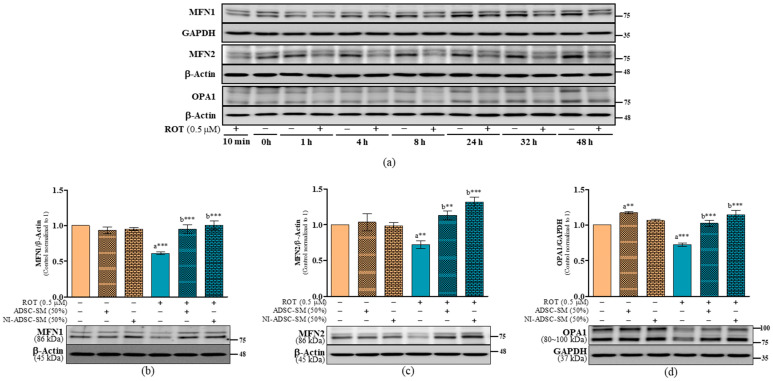
Effects of the NI-ADSC-SM on mitochondrial fusion-associated proteins. Cells treated with or without ROT (0.5 μM) at different timepoints were assessed for MFN1, MFN2, and OPA1 expression by Western blotting (**a**). Cells were incubated in the presence of ROT (0.5 μM) or DMSO for 48 h alone or with the ADSC-SM or NI-ADSC-SM (each diluted by 50%) during the last 24 h. The levels of MFN1/β-actin (**b**), MFN2/β-actin (**c**), and OPA1/GAPDH (**d**) protein expression were assessed by Western blotting. Data are shown as the means (bars) and SEM (error bars) of three independent cell culture experiments. Statistical analysis was performed using one-way ANOVA with Tukey’s post-test. Statistical comparisons: a—compared with control; b—compared with ROT; ** *p* < 0.01 and *** *p* < 0.001.

Mitochondrial fusion is the union of two mitochondria into one elongated mitochondrion, which is controlled by MFN1, MFN2, and OPA1. In the time-course study, ROT decreased the levels of MFN1, MFN2, and OPA1 at different timepoints (Figure 7a). We observed a decrease in MFN1 (*p* < 0.001; Figure 7b), MFN2 (*p* < 0.01; Figure 7c), and OPA1 (*p* < 0.001; Figure 7d) after exposure to ROT for 48 h in SH-SY5Y cells. Treatment with the NI-ADSC-SM (*p* < 0.001 for all) or ADSC-SM (*p* < 0.001 for MFN1 and OPA1; *p* < 0.01 for MFN2) at the final 24 h significantly increased the levels of MFN1, MFN2, and OPA1 in the ROT-exposed cells. Treatment with the ADSC-SM also increased the OPA1 level in control SH-SY5Y cells (*p* < 0.01; Figure 7d). 

### 2.7. Effects of the NI-ADSC-SM on Endoplasmic Reticulum Stress Protein Expression after ROT Exposure

**Figure 8:**
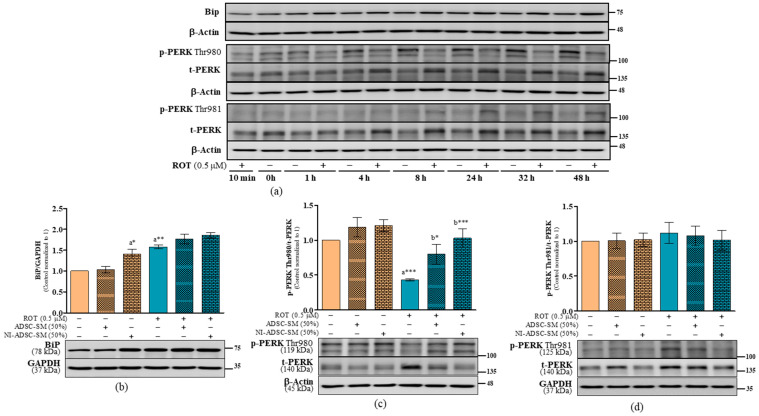
Effects of the NI-ADSC-SM on BiP and PERK expression. Cells treated with or without ROT (0.5 μM) at different timepoints were assessed for BiP and PERK by Western blotting (**a**). Cells were incubated in the presence of ROT (0.5 μM) or DMSO for 48 h alone or with the ADSC-SM or NI-ADSC-SM (each diluted by 50%) during the last 24 h. The levels of BiP/GAPDH (**b**), p-PERK Thr980/t-PERK (**c**), and p-PERK Thr981/t-PERK (**d**) protein expression were assessed by Western blotting. Data are shown as the means (bars) and SEM (error bars) of three independent cell culture experiments. Statistical analysis was performed using one-way ANOVA with Tukey’s post-test. Statistical comparisons: a—compared with control; b—compared with ROT; * *p* < 0.05, ** *p* < 0.01, and *** *p* < 0.001. p-, phosphorylated; t-, total.

**Figure 9:**
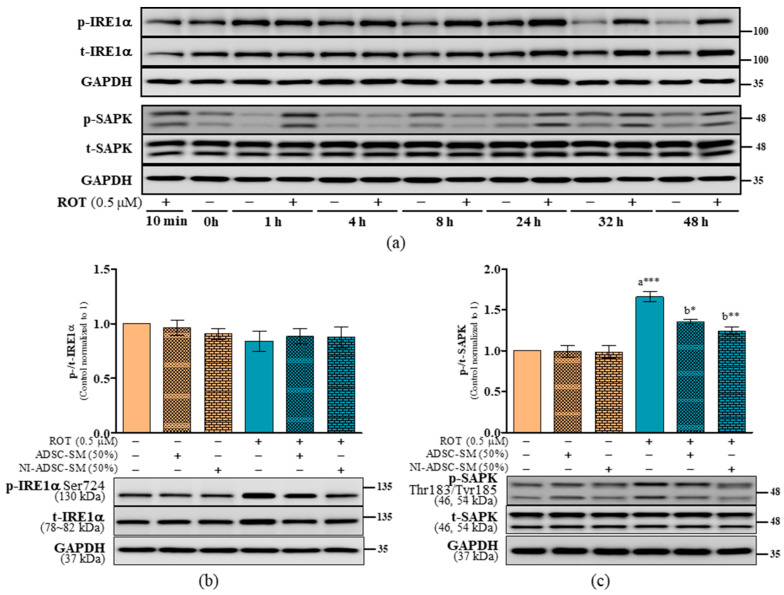
Effects of the NI-ADSC-SM on IRE1α and SAPK/JNK expression. Cells treated with or without ROT (0.5 μM) at different timepoints were assessed for IRE1α and SAPK by Western blotting (**a**). Cells were incubated in the presence of ROT (0.5 μM) or DMSO for 48 h alone or with the ADSC-SM or NI-ADSC-SM (each diluted by 50%) during the last 24 h. The levels of p-IRE1α Ser724/t-IRE1α (**b**) and p-SAPK Thr183-Tyr185/t-SAPK (**c**) protein expression were assessed by Western blotting. Data are shown as the means (bars) and SEM (error bars) of three independent cell culture experiments. Statistical analysis was performed using one-way ANOVA with Tukey’s post-test. Statistical comparisons: a—compared with control; b—compared with ROT; * *p* < 0.05, ** *p* < 0.01, and *** *p* < 0.001. p-, phosphorylated; t-, total.

The ER is a crucial organelle involved in protein production. In this present study, ROT-induced toxicity increased the ratios of BiP/GAPDH (*p* < 0.01; Figure 8b), p-PERK Thr981/GAPDH (*p* < 0.05; Figure 8e and Supplementary Figure S3c), t-PERK/GAPDH (*p* < 0.05; Figure 8e and Supplementary Figure S3d), t-PERK/β-actin (*p* < 0.05; Figure 7d and Supplementary Figure S3b), p-IRE1α Ser724/GAPDH (*p* < 0.01; Figure 9b and Supplementary Figure S4a), t-IRE1α/GAPDH (*p* < 0.01; Figure 9b and Supplementary Figure S4b), and p-SAPK Thr183,Tyr185/GAPDH (*p* < 0.001; Figure 9c and Supplementary Figure S4c), while decreasing the ratios of p-PERK Thr980/t-PERK (*p* < 0.001; Figure 8c) and p-PERK Thr980/β-actin (*p* < 0.001; Supplementary Figure S3a). Treatment with NI-ADSC-SM after ROT exposure did not modify BiP levels; however, BiP increased when treated in control SH-SY5Y cells (*p* < 0.05; Figure 8b). NI-ADSC-SM treatment decreased the levels of p-PERK Thr981/GAPDH (*p* < 0.05; Supplementary Figure S3c and Figure 8d), t-PERK (*p* < 0.05; Supplementary Figure S3b,d), p-IRE1α Ser724 (*p* < 0.01; Figure 9b and Supplementary Figure S4a), t-IRE1α (*p* < 0.01; Figure 9b and Supplementary Figure S4b), and p-SAPK Thr183-Tyr185 (*p* < 0.05; Figure 9c and Supplementary Figure S4c). Treatment with the NI-ADSC-SM or ADSC-SM increased the expression of p-PERK Thr980 (*p* < 0.001 by NI-ADSC-SM; *p* < 0.05 by ADSC-SM; Figure 8c and Supplementary Figure S3a).

**Figure 10:**
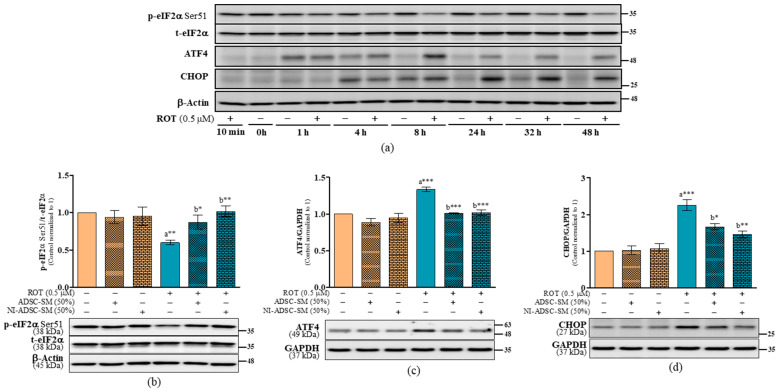
Effects of the NI-ADSC-SM on eIF2α, ATF4, and CHOP expression. Cells treated with or without ROT (0.5 μM) at different timepoints were assessed for above protein expressions by Western blotting (**a**). Cells were incubated in the presence of ROT (0.5 μM) or DMSO for 48 h alone or with the ADSC-SM or NI-ADSC-SM (each diluted by 50%) during the last 24 h. The levels of p-eIF2α Ser51/t-eIF2α (**b**), ATF4/GAPDH (**c**), and CHOP/GAPDH (**d**) protein expression were assessed by Western blotting. Data are shown as the means (bars) and SEM (error bars) of three independent cell culture experiments. Statistical analysis was performed using one-way ANOVA with Tukey’s post-test. Statistical comparisons: a—compared with control; b—compared with ROT; * *p* < 0.05, ** *p* < 0.01, and *** *p* < 0.001. p-, phosphorylated; t-, total.

As seen in Figure 10a, the expression of p-eIF2α at Ser51 was decreased, but the levels of ATF4 and CHOP were increased by ROT in the time-dependent toxicity study. p-eIF2α Ser51 was significantly decreased (*p* < 0.01; Figure 10b and Supplementary Figure S5a), while ATF4 (*p* < 0.001; Figure 10c) and CHOP (*p* < 0.001; Figure 10d) were increased by ROT after 48 h. NI-ADSC-SM treatment increased p-eIF2α Ser51 (*p* < 0.01; Figure 10b and Supplementary Figure S5a) and decreased ATF4 (*p* < 0.001; Figure 10c) and CHOP (*p* < 0.01; Figure 10d) after ROT exposure. Treatment with the ADSC-SM showed comparably less protective effect to ROT toxicity than the NI-ADSC-SM. Treatment with ROT, the ADSC-SM, or the NI-ADSC-SM alone or combined did not change the total levels of eIF2α in SH-SY5Y cells (Supplementary Figure S5b).

### 2.8. Effects of the NI-ADSC-SM on IP3R-GRP75-VDAC Tethering Protein Expression after ROT Exposure

**Figure 11:**
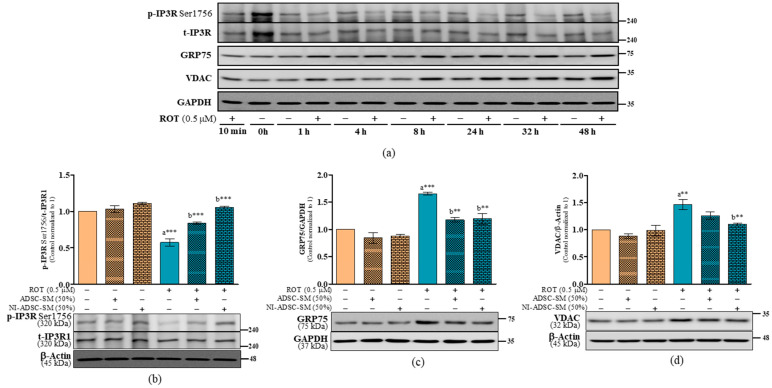
Effects of the NI-ADSC-SM on ER–mitochondrial signaling proteins. Cells treated with or without ROT (0.5 μM) at different timepoints were assessed for IP3R1, GRP75, and VDAC expression by Western blotting (**a**). Cells were incubated in the presence of ROT (0.5 μM) or DMSO for 48 h alone or with the ADSC-SM or NI-ADSC-SM (each diluted by 50%) during the last 24 h. The levels of p-IP3R Ser1756/t-IP3R1 (**b**), GRP75/GAPDH (**c**), and VDAC/β-actin (**d**) protein expression were assessed by Western blotting. Data are shown as the means (bars) and SEM (error bars) of three independent cell culture experiments. Statistical analysis was performed using one-way ANOVA with Tukey’s post-test. Statistical comparisons: a—compared with control; b—compared with ROT; ** *p* < 0.01 and *** *p* < 0.001. p-, phosphorylated; t-, total.

It is interesting to understand mitochondria, the ER, and their interactions in NDD that regulate Ca^2+^ transfer between these organelles. IP3R1 has been shown to be relevant to ER–mitochondria Ca^2+^ coupling by forming a complex with VDAC1 and GRP75. We first studied the expression of the p-IP3R at Ser1756, t-IP3R1, GRP75, and VDAC in different timepoints of ROT toxicity (Figure 11a). p-IP3R Ser1756 was decreased (*p* < 0.001; Figure 11b and Supplementary Figure S5c), whereas GRP75 (*p* < 0.001; Figure 11c) and VDAC (*p* < 0.01; Figure 11d) were increased by ROT toxicity in SH-SY5Y cells. ROT, the ADSC-SM, and the NI-ADSC-SM did not alter the levels of t-IP3R1 (Figure 11b and Supplementary Figure S5d). In contrast, treatments with the ADSC-SM or NI-ADSC-SM increased p-IP3R Ser1756 (*p* < 0.001; Figure 11b and Supplementary Figure S5c) and decreased GRP75 levels (*p* < 0.01; Figure 11c). ADSC-SM treatment did not modify VDAC, but the NI-ADSC-SM decreased the VDAC levels after exposure to ROT (*p* < 0.01; Figure 11d).

## 3. Discussion

ROT easily crosses biological membranes due to its high lipophilicity and inhibits complex I, which can reproduce pathological conditions of PD-like symptoms, including aggregation of α-syn [15]. LRRK2 is a ubiquitously expressed, large homodimeric protein that acts as a hub for multiprotein signaling and participates in protein–protein interactions, cytoskeletal dynamics, mitochondrial function, and autophagy [22]. This cytoplasmic protein may associate with intracellular membranes, such as the OMM, Golgi apparatus, and ER [23]. ROT increased LRRK2 levels in the Triton X-100-soluble and -insoluble cell lysates in this study, coinciding with another study suggesting that ROT-induced LRRK2 activation leads to an overall reduction of protein translation [24]. An increase in LRRK2 can disrupt its normal physiological functions, which results in synaptic dysfunction [25], increased mitochondrial Ca^2+^ uptake [26], and deregulation of autophagy through lysosomal degradation [27]. NI-ADSC-SM treatment inhibited the ROT-induced increase of LRRK2 in SH-SY5Y cells in this study, which also supports that LRRK2 inhibition could prevent the loss of dopaminergic neurons [28]. Therefore, inhibiting LRRK2 is potentially beneficial in PD.

Mitophagy, the selective degradation of mitochondria by autophagy, is essential for maintaining neuronal health by degrading and recycling cellular material. PINK1 is a protein kinase required to recruit parkin and Ub to damaged mitochondria to initiate mitophagy on the OMM [29,30]. In this present study, the levels of PINK1 and parkin were decreased by ROT in SH-SY5Y cells. This result suggests that the degradation of cytosolic PINK1 by the Ub–proteasome system led to low levels of PINK1 [31]. Studies also reported that the loss of PINK1 or parkin in SH-SY5Y cells induced higher mitochondrial fragmentation facilitated by DRP1 [7,32]. Loss of parkin can cause uncoupling of mitochondria and the ER and a decrease in MAM under mitophagy induction [33]. The depletion of PINK1 or parkin increases ROS-induced apoptotic cell death [34]. These results suggest that activation of mitophagy could recruit ubiquitinated substrates, such as misfolded α-syn protein aggregates, for clearance [16].

The ubiquitination of mitochondria under oxidative stress accumulates aggregation of misfolded proteins, which are recognized by the autophagic adaptor protein p62 (SQSTM1; sequestome1) and cleared through the mitophagy [35]. ROT-induced toxicity downregulated the autophagic clearance [16], suggesting that the protein aggregates may accumulate with increased levels of Ub conjugates [36]. In this present study, ROT toxicity increased the Ub (monomer and polyubiquitinated) in the Triton X-100-insoluble cell lysates; however, it decreased the Triton X-100-soluble Ub. Studies indicate that phosphorylated α-syn is ubiquitinated [37], which further enhances cellular dysfunction [38]. p-S129-α-syn was increased in the Triton X-100-insoluble fractions [15], reflecting the current results that ubiquitination attempts to target misfolded proteins for degradation. In this present study, NI-ADSC-SM treatment increased parkin and decreased insoluble Ub expression, suggesting the neuroprotective potential against ROT toxicity. The NI-ADSC-SM did not recover PINK1 levels in this study, suggesting that PINK1 has other distinct and uncharacterized functions [6]. However, overexpression of parkin was able to rescue the α-syn-induced toxicity associated with PD [39].

Several substrates of OMM proteins have been ubiquitinated by the PINK1/parkin-mediated signaling pathway [40]. DJ-1 is almost ubiquitously expressed in the brain and is present in synaptic terminals, mitochondria, and membranous organelles [41]. DJ-1 colocalizes with LBs and can downregulate α-syn by forming an E3 ligase complex with PINK1/parkin [42]. The decreased levels of DJ-1 after ROT exposure seen in this present study may be attributed to increased ROS production [43]. α-syn aggregation is promoted by the loss of DJ-1 via the increased degradation of LAMP2 [44]. A decrease in DJ-1 in MAMs alters Ca^2+^ transfer [33], leading to mitochondrial dysfunction [45]. ROT toxicity decreased the LAMP2 levels and increased the oligomerization of α-syn in SH-SY5Y cells. Treatment with the NI-ADSC-SM increased DJ-1 in this study. NI-ADSC-SM also increased the LAMP2 levels and decreased the α-syn oligomerization in our previous results [15,16].

Mitochondrial proteins positioned within the matrix cooperate with the TOM complex via the OMM receptor TOM20 [46]. PINK1 is imported through the TOM complex in healthy mitochondria [47], while parkin ubiquitinates multiple substrate proteins, including TOM20 [40]. In PD, p-Ser129-α-syn binds to TOM20, impairing mitochondrial function [48]. In this study, TOM20 levels were decreased by ROT, whereas the NI-ADSC-SM increased TOM20 to above-normal levels. Studies reported that oligomeric, but not monomeric, α-syn binds to TOM20 causing mitochondrial dysfunction [48]. However, increased TOM20 levels in this study may be due to the reduction of oligomeric α-syn as reported earlier [15] which imports mitochondrial precursor proteins, increasing mitochondrial function [49].

Mitochondria undergo constant fission and fusion sequentially, and these functions rely on the levels of DRP1 phosphorylation and Mfn1 and 2 and OPA1 expression [50]. DRP1 is essential for the mitochondrial distribution in axons, dendrites, and synapses. However, phosphorylation of DRP1 at Ser616 activates DRP1, which promotes translocation from the cytosol to the OMM, inducing mitochondrial division and fragmentation [51]. Phosphorylation at Ser637 of DRP1 inhibits DRP1 activity, thus preventing mitochondrial fragmentation and regulating mitochondrial morphology [52]. The interplay between Ser616 and Ser637 via the PINK1/parkin pathway can drive mitophagy [51]. In this present study, p-DRP1 Ser616 was significantly increased, while p-DRP1 Ser637 was decreased by ROT. LRRK2 overexpression increases mitochondrial fragmentation and clearance by interacting with DRP1 [53]. Moreover, we found that NI-ADSC-SM treatment could almost completely inhibit the ROT-induced increase in p-DRP1 Ser616 and activate p-DRP1 Ser637, suggesting that fragmented mitotic mitochondria can escape from apoptotic cell death via mitophagy [51].

Mitochondrial fusion is controlled by MFN1 and MFN2 localized to the OMM and OPA1 located in the IMM [8]. MFN2 was reported to tether the ER to the mitochondria by directly interacting with either MFN1 or MFN2 on the OMM [54] to regulate mitochondrial Ca^2+^ uptake from the ER [55]. PINK1/parkin can regulate MAMs through MFN2 [56]. OPA1 has been shown to be responsible for the fusion of the IMM associated with cristae folding and regulating the respiratory chain supercomplex assembly [57]. Depletion of OPA1 during apoptosis causes mitochondrial fragmentation and modifies the shape of the cristae [58]. In this present study, the low levels of MFN1, MFN2, and OPA1 during ROT-induced toxicity suggest that mitochondrial fusion is repressed, leading to the marked accumulation of damaged mitochondria. Abnormally high mitochondrial fission induces ROS production to activate PINK/parkin-dependent mitophagy [59], uncoupling the mitochondria from the ER via the degradation of MFN2 [6]. Lowered MFN2 levels decreased the distance between the ER and OMM to impair Ca^2+^ uptake into the mitochondria [60]. These results suggest that ROT toxicity promotes mitochondrial fission while inhibiting mitochondrial fusion in SH-SY5Y cells. As expected, NI-ADSC-SM treatment increased these fusion proteins after exposure to ROT is likely sufficient to rescue mitochondrial dysfunction-associated pathologies.

The ER controls posttranslational protein processing and transport; however, the accumulation of misfolded proteins is upregulated during ER dysfunction [61]. PERK, IRE1α, and ATF6 are associated with BiP/GRP78 in normal conditions but are released during ERS, triggering the unfolded protein response (UPR) [62] to recover protein homeostasis or induce apoptosis [63]. In this present study, BiP, p-Thr981 PERK, t-PERK, p-IRE1α, and t-IRE1α were increased, while p-Thr980 PERK was decreased during ROT toxicity. These results showed that ROT-induced ERS led to the disassociation of BiP from the luminal domains of both PERK and IRE1α, enabling auto-phosphorylation [64]. Accumulation of unfolded proteins activates IRE1α at Ser724, leading to apoptosis through stress-activated protein kinase (SAPK; c-Jun N-terminal kinase; JNK) signaling during ERS [11]. A rapid increase in p-SAPK at Thr183/Tyr185 upon ROT toxicity amplifies ERS subsequently activates pro-apoptotic cell death. Activated SAPK enters the nucleus and promotes cell death associated with CHOP transcription [65]. However, the NI-ADSC-SM reverted the changes on PERK, IRE1α, and SAPK induced by ROT. In addition, the NI-ADSC-SM did not change BiP levels after exposure to ROT, and NI-ADSC-SM-treated control cells showed increased BiP levels in this study. Another study showed that BiP was increased to protect cells from oxidative stress. Thus, BiP can momentarily bind to hydrophobic residues on proteins to refold or prevent aggregation [66].

PERK activation blocks the entrance of synthesized proteins into the ER [67], thus inactivating the global protein translation initiation key target eIF2α, which causes the destruction of protein translation and dropping of ER protein load [68]. In this present study, ROT decreased p-Ser51 eIF2α levels, while the NI-ADSC-SM increased them in SH-SY5Y cells. Dephosphorylation of eIF2α by ROT may halt protein synthesis [69]. We suggest that increased p-eIF2α by the NI-ADSC-SM may upregulate basal autophagy, antioxidant responses, and amino acid metabolism in the UPR [63]. These results coincide with other studies suggesting that p-eIF2α may be protective [70,71]. These protective responses result in enhanced protein degradation and subsequently increase the ER protein folding capacity [61]. Induction of the transcription factors ATF4 and CHOP by ERS is dependent on PERK [72] and is also evidenced in this study during ROT toxicity. When the UPR pathway is compromised and can no longer restore ER homeostasis, PERK induces CHOP to stimulate ERS-dependent cell death [73]. ATF4 also controls the expression of pro-apoptotic CHOP [74]. ATF4 expression in the axon triggers a cascade [75]. CHOP-promoted cell death in PD has been linked to increased ROS and decreased Bcl-2 [2].

ERS includes the release of Ca^2+^ from ER stores and the physical interaction of the ER and mitochondria [76]. Ca^2+^ is required inside the mitochondria for the production of ATP. ER–mitochondria tethering regulates Ca^2+^ homeostasis, lipid transfer, mitochondrial dynamics, and autophagy [29]. LRRK2 mutations have been linked to impaired autophagic regulation through altered ER and lysosomal Ca^2+^ signaling pathways [77]. Ca^2+^ exchange between the ER and mitochondria [13] is mediated by a molecular tripartite tethering complex, IP3R-GRP75-VDAC [14], in the MAM. IP3R, responsible for Ca^2+^ release from the ER, interacts with VDAC1 at the OMM via a chaperone, GRP75 [29]. In this present study, ROT-induced toxicity decreased the p-Ser1756 IP3R while increasing the levels of GRP75 and VDAC. ROT also disrupts intracellular Ca^2+^ homeostasis [61]. IP3R plays important roles in protecting cells against apoptosis [54], and high Ca^2+^ concentrations can inhibit IP3R [78] function, leading to apoptosis by depressing the mitochondrial membrane potential [79].

Loss of IP3R activity activates AMP-activated protein kinase (AMPK), which in turn inhibits mammalian target of rapamycin (mTOR) signaling [80], which was also reported in our previous publication [16]. Impaired tethering of the ER and mitochondria can be mediated by altered proteins involved in MAMs. Upregulated GRP75 and VDAC expression is crucial for the increased Ca^2+^ load, leading to mitochondrial dysfunction in neurons [81] and a higher susceptibility for cell death [54]. In addition, PD-associated α-syn mutations decrease ER–mitochondria connections [82]. NI-ADSC-SM treatment increased p-Ser1756 IP3R and inhibited the expression of GRP75 and VDAC during ROT toxicity. The increased anti-apoptotic protein Bcl-2 upregulates the phosphorylation of IP3R and lowers pro-apoptotic ER–mitochondrial Ca^2+^ fluxes [83], suggesting that IP3R is central for tethering mitochondria close to the ER [84].

## 4. Materials and Methods

### 4.1. Secretome-Containing Culture Medium Collection from Primary ADSCs and Neural-Induced ADSCs

Adipose tissues from human donors were attained according to the Ethics Committee of Chonnam National University Medical School (IRB: I-2009-03-016). Primary ADSCs were isolated, and adherent cells were grown at 37 °C in a humidified incubator (5% CO_2_/95% air) with Dulbecco’s modified Eagle’s medium (DMEM; Hyclone, Logan, UT, USA) containing 10% fetal bovine serum (FBS, Hyclone), 1% penicillin–streptomycin (Gibco BRL, Grand Island, NY, USA), and 0.2% amphotericin B (Gibco). Approximately 80% confluence of the primary ADSCs (passages 3–5) were maintained in DMEM supplemented with reduced FBS at 1% for seven days. The secretome-containing culture medium from primary ADSCs (ADSC-SM) was collected, pooled, filtered with a sterile 0.2 μm syringe filter, and kept at −80 °C until used for treatment. For the neural-induced secretome, primary ADSCs (passages 3–5) cultured in DMEM supplemented with 1% FBS were supplemented with 100 ng/mL bFGF (Invitrogen Co., Carlsbad, CA, USA) for the first seven days and further incubated for another seven days with 10 μM forskolin (Sigma Chemical Co., St. Louis, MO, USA) as per our previous studies [19,20,21]. The neural-induced secretome-containing culture medium (NI-ADSC-SM) was collected without NI-ADSCs, pooled, filtered using a sterile 0.2 μm syringe filter, and kept at −80 °C until used for treatment. Several batches of the ADSC-SM and NI-ADSC-SM were collected from multiple cell cultures and neural induction for the consequent experiments.

### 4.2. Cell Culture

The human neuroblastoma cell line SH-SY5Y (RRID: CVCL_0019; ATCC^®^ CRL-2266; American Type Culture Collection, Manassas, VA, USA) was cultured with 10% FBS and 1% penicillin–streptomycin supplemented DMEM (Welgene Inc., Gyeongsan, Republic of Korea) at 37 °C in a humidified incubator containing 5% CO_2_/95% air. Confluent cultures from passages 15–22 were used for experiments. Briefly, cultured cells were rinsed with phosphate-buffered saline (PBS), dissociated with 0.25% trypsin–EDTA solution, then reseeded at an equal density of 50,000 cells/mL in DMEM with 1% FBS, and kept for overnight before being used for the experiments.

### 4.3. Rotenone Preparation

A ROT (R8875, Sigma) stock solution at 10 mM in a polar aprotic solvent, dimethyl sulfoxide (DMSO; D2650, Sigma), was aliquoted and kept at −80 °C and used for experiments within six months. A ROT working solution (250 μM) was prepared with DMEM (without FBS) for each experiment. The remaining working solution diluted from the stock solution was discarded.

### 4.4. ROT Toxicity and ADSC-SM and NI-ADSC-SM Treatments

Time-dependent effects of ROT (0.5 μM)-induced toxicity on SH-SY5Y cells were assessed to characterize the protein signaling pathway changes (Supplementary Figure S1a). SH-SY5Y cells were incubated for 24 h in the presence of 0.5 μM ROT or DMSO (control). The cell culture medium was collected with floating cells, centrifuged at 3000 rpm for three minutes, and the supernatant was discarded. The pelleted cells were resuspended in a fresh medium and added to their respective culture plate. Either the ADSC-SM or NI-ADSC-SM (each diluted at 50% in DMEM with 1% FBS) was added, and the cells were incubated in the presence of 0.5 μM ROT or DMSO for another 24 h (Supplementary Figure S1b). FBS at 1% was maintained throughout the study. Several sets of experiments were performed with different passages of SH-SY5Y cells treated with multiple sets of the ADSC-SM or NI-ADSC-SM against ROT-induced toxicity.

### 4.5. Estimation of Intracellular Calcium (Ca^2+^) by Fura-2AM

Fura-2AM (1 mM; F-1221, Molecular Probes, Carlsbad, CA, USA) was added and incubated for one hour at 37 °C in a dark incubator. The fura-2AM-containing cell culture medium was then removed, the cells were washed twice with fresh DMEM (without FBS), and a suspension was prepared. Fluorescence was measured using a SpectraMax M2 fluorescence spectrometer (Molecular Devices, Sunnyvale, CA, USA) by SoftMax Pro 5.4.6 software (Molecular Devices) with excitations at 320 nm and 355 nm and emission at 538 nm. The levels of Ca^2+^ were calculated by the ratio of 320/355 nm excitation and expressed as a percentage of the control. The assay was performed in triplicate.

### 4.6. Preparation of Triton X-100-Soluble and -Insoluble Fractions and Western Blotting

Detached and adherent cells were collected by scraping and centrifugation before being washed with PBS. Then, the cells were immersed with Triton X-100-soluble cell lysis buffer consisting of rapid immunoprecipitation assay (RIPA) buffer (#89901, Thermo Scientific, Rockford, IL, USA), Halt protease inhibitor cocktail (#87789, Thermo Scientific), Halt phosphatase inhibitor cocktail (#78420, Thermo Scientific), and 1% Triton X-100 (X100, Sigma). The cells were incubated for 30 min on ice at 8 °C. Thereafter, the lysates were centrifuged at 13,200 rpm (16,000× *g*) for 20 min at 4 °C, and the cell lysate supernatants (Triton X-100-soluble fractions) were collected. The remaining cell pellets were washed with PBS, dissolved in a Triton X-100-insoluble cell lysis buffer consisting of 2% sodium dodecyl sulfate (SDS, L3771, Sigma) and Triton X-100-soluble cell lysis buffer, and sonicated for one minute on ice at intervals of 10 s (Triton X-100-insoluble fractions). The BCA Protein Assay Kit (#23225, Thermo Scientific) was used to estimate the protein levels, and equal amounts (10 μg) were loaded on 5–12% SDS–polyacrylamide gels. The proteins were separated according to their molecular weight in the gels and were transferred onto polyvinylidene difluoride (PVDF) membranes (IPVH00010, Millipore, Bradford, MA, USA). The membranes were blocked with 5% nonfat dried milk or 1% bovine serum albumin (BSA) dissolved in the washing buffer (TBS-T; Tris-buffered saline, pH 7.6 containing 0.1% Tween 20). The membranes were then incubated with primary and secondary antibodies. The antibodies used (acquired from Abcam, Cambridge, MA, USA; Biorbyt, Cambridge, UK; Cell Signaling Technology Inc., Danvers, MA, USA; and Santa Cruz Biotechnology, Santa Cruz, CA, USA) are listed in Supplementary Table S1. Lastly, the bands were visualized by an enhanced chemiluminescence (ECL) system (WBLUR0500, Millipore, Billerica, MA, USA) and a luminescent image analyzer (LAS 4000, GE Healthcare, Little Chalfont, UK). After imaging the phosphorylated proteins, the membranes were stripped with Western Blot Stripping Buffer (#21059, Thermo Scientific) and subsequently used to detect total protein forms. β-actin or GAPDH were used to normalize the target protein levels. Phospho-protein signals were normalized against the total (non-phosphorylated) forms of the same target protein. ImageJ software (National Institutes of Health, Bethesda, MD, USA) was used for densitometric analysis.

### 4.7. Statistical Analysis

Data are shown as the mean ± standard error of the mean (SEM) of three independent cell culture experiments. Microsoft Excel and GraphPad Prism^®^ 5.0 software (GraphPad Software Inc., San Diego, CA, USA) were used for data processing, analyzing statistical comparisons, and preparing the bar charts. One-way analysis of variance (ANOVA) followed by Tukey’s post hoc multiple-comparison tests were performed, and *p*-values of less than 0.05 were considered statistically significant for toxicity or treatment groups.

## 5. Conclusions

**Figure 12:**
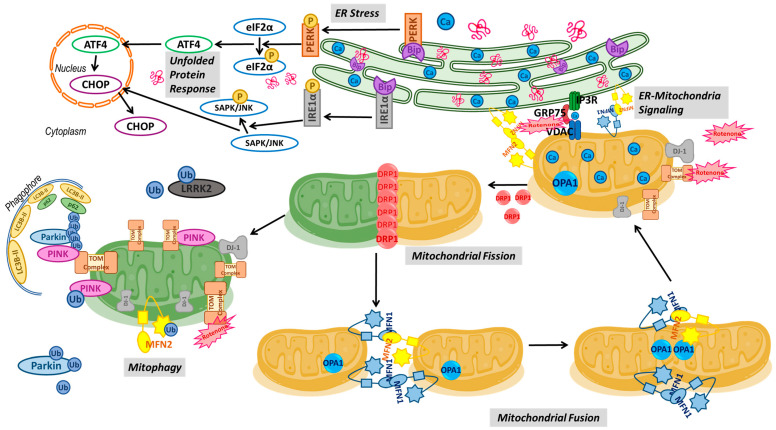
Schematic representation of the proposed mechanisms of the NI-ADSC-SM in response to ROT toxicity.

ROT-induced toxicity in SH-SY5Y cells resulted in impaired cellular homeostasis of mitochondria, the ER, and MAM tethering proteins. Additionally, increased cell-death-associated Ca^2+^ is crucial for understanding the pathogenesis of PD. Increased LRRK2 disrupts PINK1/parkin-dependent mitophagy, and mitochondrial fusion target increased mitochondrial fission. In addition, misfolded proteins during ERS induced by ROT toxicity triggered the release of PERK and IRE1α from BiP, promoting the activation of ATF4, CHOP, and SAPK. Increased expression of GRP75 and VDAC may be accompanied by mitochondrial Ca^2+^ overload and reduced IP3R tethering between the ER and mitochondria. NI-ADSC-SM treatment attenuated the ROT-induced dysfunction in mitochondria and the ER. Taken together, our findings may help uncover the molecular mechanisms of ROT-induced neurotoxicity contributing to the signaling pathways of mitochondria, the ER, and their interaction in NDD (Figure 12). Therefore, the “secretome” released during neural differentiation from MSCs into the conditioned medium may have a vital role in treating NDDs. Thus, the NI-ADSC-SM is suggested to have therapeutic potential through the various biological molecules released during neural differentiation and may be sufficient to rescue pathologies in PD.

## Data Availability

The data presented in this study are available in the article and Supplementary Materials.

## Supplementary Materials

The following supporting information can be downloaded at https://www.mdpi.com/article/10.3390/ijms24065622/s1.
