# Supplementary material for: Neuroprotective Effects of the Neural-Induced Adipose-Derived Stem Cell Secretome against Rotenone-Induced Mitochondrial and Endoplasmic Reticulum Dysfunction
Source: Int J Mol Sci. 2023 Mar 15;24(6):5622. doi: 10.3390/ijms24065622 (PMC10054666; doi:10.3390/ijms24065622)
Supplement: Supplementary file 1 [file ijms-24-05622-s001.zip › ijms-2235542-supplementary.pdf]

Supplementary materials to

Article

Neuroprotective Effects of the Neural-induced Adipose-derived Stem Cell Secretome Against Rotenone-induced Mitochondrial and Endoplasmic Reticulum Dysfunction

Mahesh Ramalingam [1,*,†], Sujeong Jang [1,†], Jinsu Hwang [1], Boeun Kim[2], Hyong-Ho Cho [3], Eungpil Kim [4] and Han-Seong Jeong [1,*]

[1] Department of Physiology, Chonnam National University Medical School, Hwasun, Jeollanam-do 58128, Korea; sujeong.jjang@gmail.com (S.J.); wlstn0128@naver.com (J.H.)

[2] Gwangju Alzheimer's Disease and Related Dementias (GARD) Cohort Center, Chosun University, Gwangju 61452, Korea; okwoosan@naver.com

[3] Department of Otolaryngology-Head and Neck Surgery, Chonnam National University Hospital, Chonnam National University Medical School, Gwangju 61469, Korea; victocho@jnu.ac.kr

[4] Biopharmaceutical Research Center, Jeonnam Bioindustry Foundation, Hwasun, Jeollanam-do 58141, Korea; keungpil@gmail.com

* Correspondence: ramalingammahesh@jnu.ac.kr (M.R.); jhsjeong@hanmail.net (H.-S.J.)

† These authors contributed equally to this work.

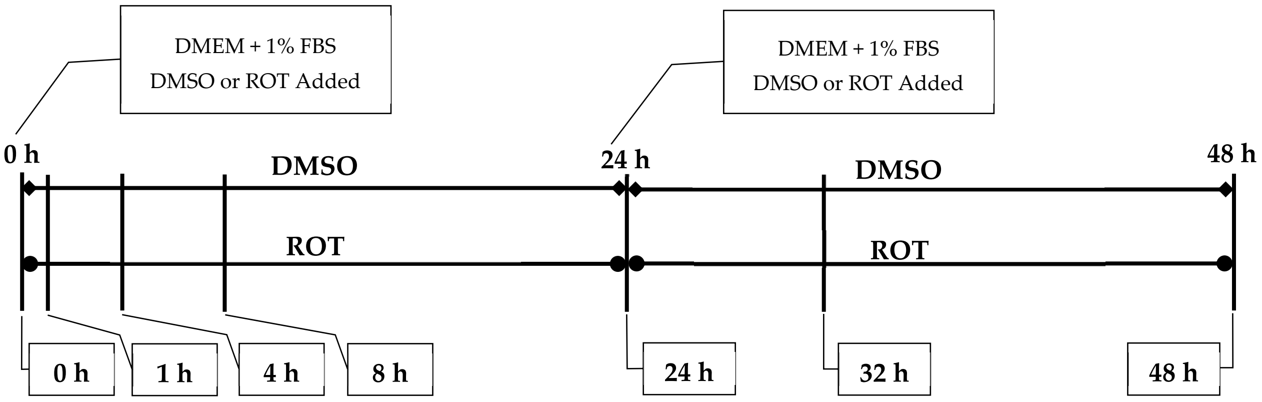

(a)

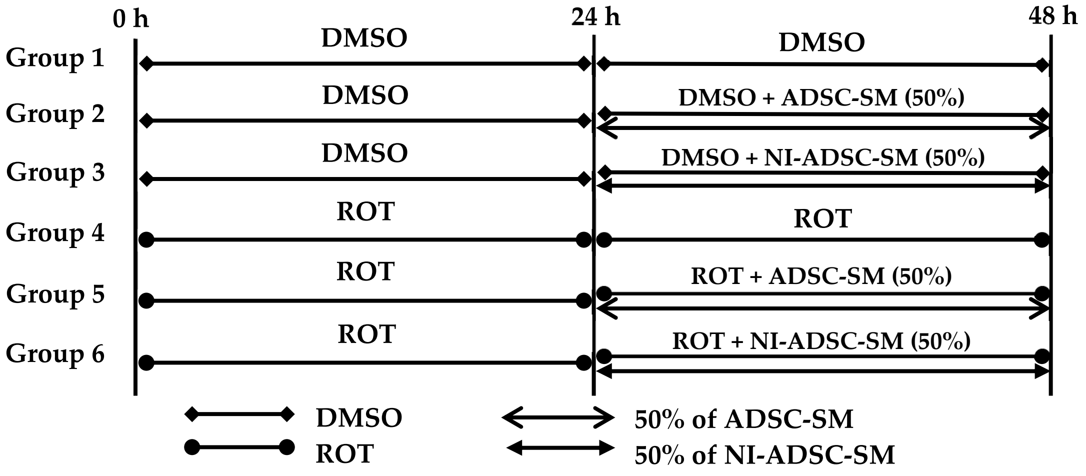

(b)

Supplementary Figure S1. (a) The experimental study plan for time-course ROT toxicity. SH-SY5Y cells were seeded at a density of 50,000 cells/mL in DMEM containing 1% FBS and incubated overnight before experiments. Cells were incubated in the presence of ROT (0.5 µM) or DMSO up to 48 h. At each time point, floating cells in cell culture medium were harvested and combined with adherent cells. Then, cells were washed with PBS and used to prepare cell lysates for Western blotting. (b) To test the therapeutic effects of NI-ADSC-SM, SH-SY5Y cells were first treated with ROT or DMSO for 24 h. The cell culture media were removed, floating cells were pelleted from the medium, the cell pellet was resuspended in the fresh medium, and added to respective wells. Then, cells were treated with or without ADSC-SM or NI-ADSC-SM at 50% dilution in DMEM supplemented with 1% FBS and incubated in the absence or presence of ROT (0.5 µM) for another 24 h. Floating cells in medium were combined with adherent cells harvested by scraping, pelleted, and washed twice with PBS. Then, cells were used to prepare cell lysates as Triton X-100-soluble and Triton X-100-insoluble fractions for Western blotting. Different passages of SH-SY5Y cells treated with different batches of ADSC-SM or NI-ADSC-SM for three independent experiments.

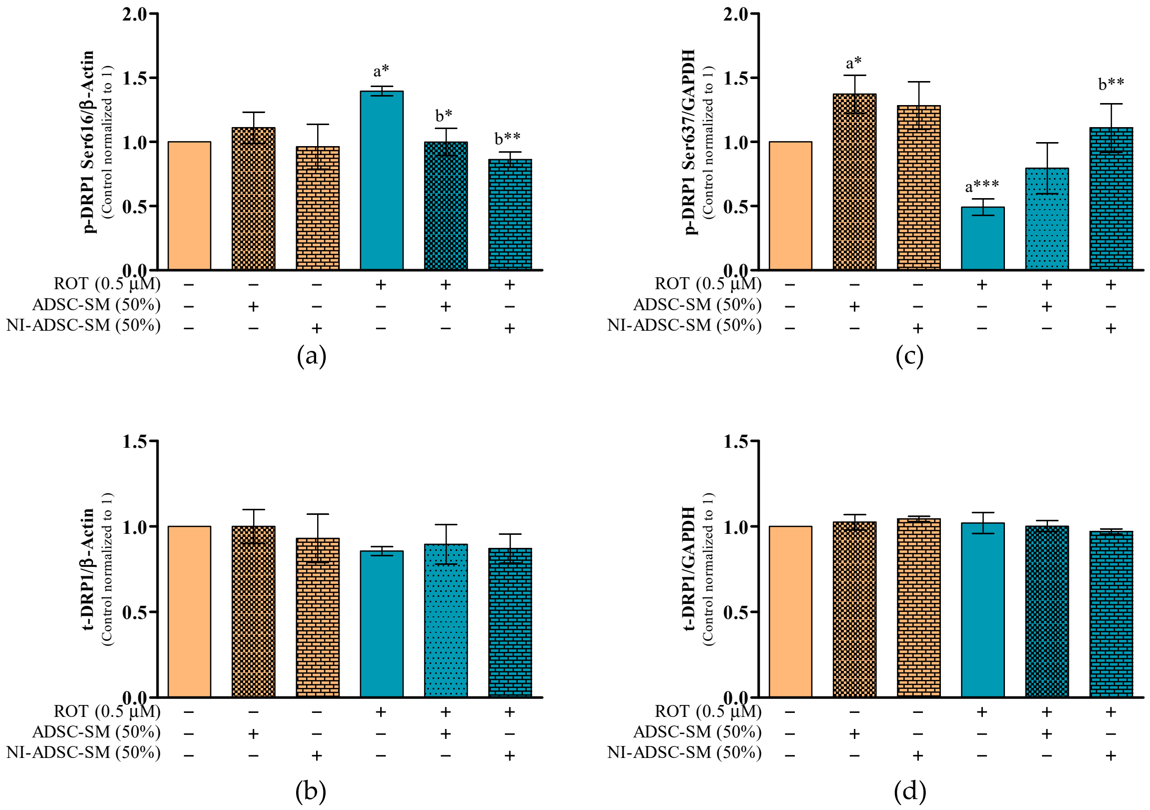

Supplementary Figure S2. SH-SY5Y cells were seeded as 50,000 cells/mL in DMEM containing 1% FBS and incubated overnight before experiments. Cells with the presence of ROT (0.5 µM) or DMSO for 48 h were treated with ADSC-SM (50%) or NI-ADSC-SM (50%) during the last 24 h. The bar graphs represent the ratio for p-DRP1 Ser616/β-actin (a), t-DRP1/β-actin (b), pDRP1 Ser637/GAPDH (c), and t-DRP1/GAPDH (d) by Western blotting. Data are shown as means (bars) and SEM (error bars) of three independent cell culture experiments, and were analyzed by one-way ANOVA with Tukey's post-test. Statistical comparison: a, compared with control; b, compared with ROT; * $p < 0.05$, ** $p < 0.01$, and *** $p < 0.001$.

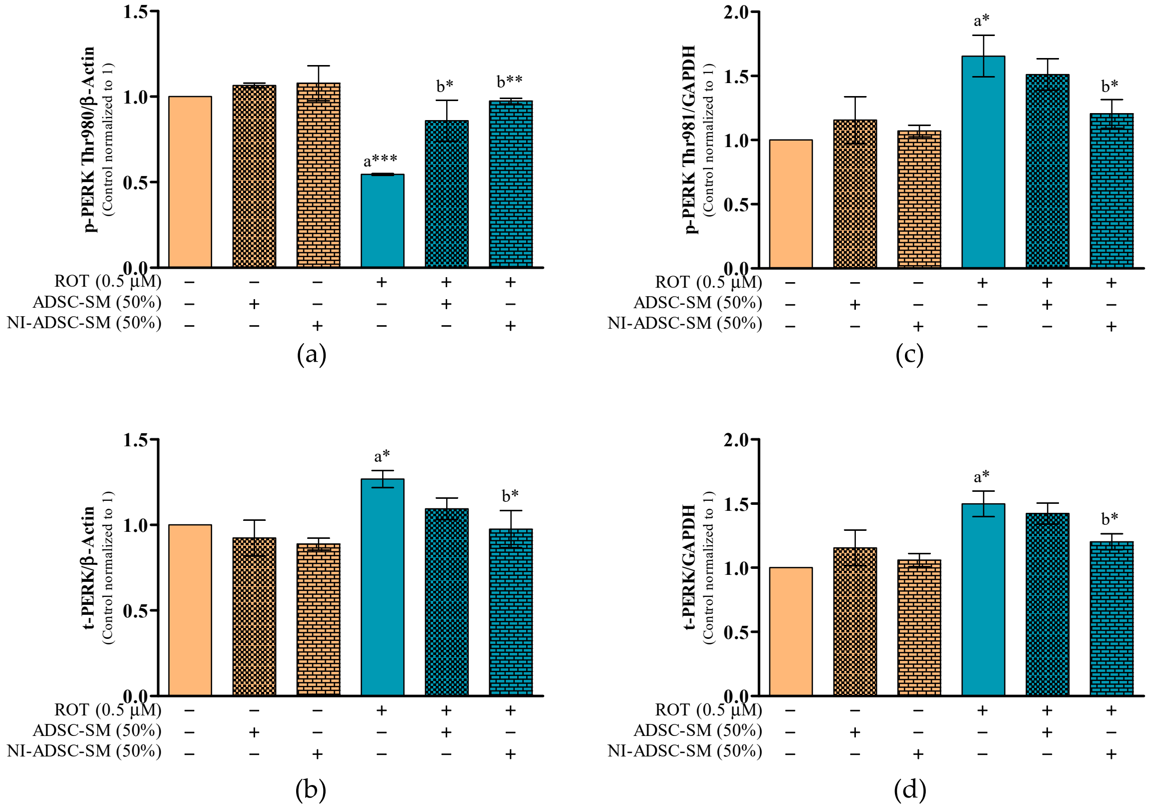

Supplementary Figure S3. SH-SY5Y cells were seeded as 50,000 cells/mL in DMEM containing 1% FBS and incubated overnight before experiments. Cells with presence of ROT (0.5 μM) or DMSO for 48 h were treated with ADSC-SM (50%) or NI-ADSC-SM (50%) during the last 24 h. The bar graphs represent the ratio for p-PERK Thr980/β-actin (a), t-PERK/β-actin (b), p-PERK Thr981/GAPDH (c), and t-PERK/GAPDH (d) by Western blotting. Data are shown as means (bars) and SEM (error bars) of three independent cell culture experiments, and were analyzed by one-way ANOVA with Tukey's post-test. Statistical comparison: a, compared with control; b, compared with ROT; * $p < 0.05$, ** $p < 0.01$, and *** $p < 0.001$.

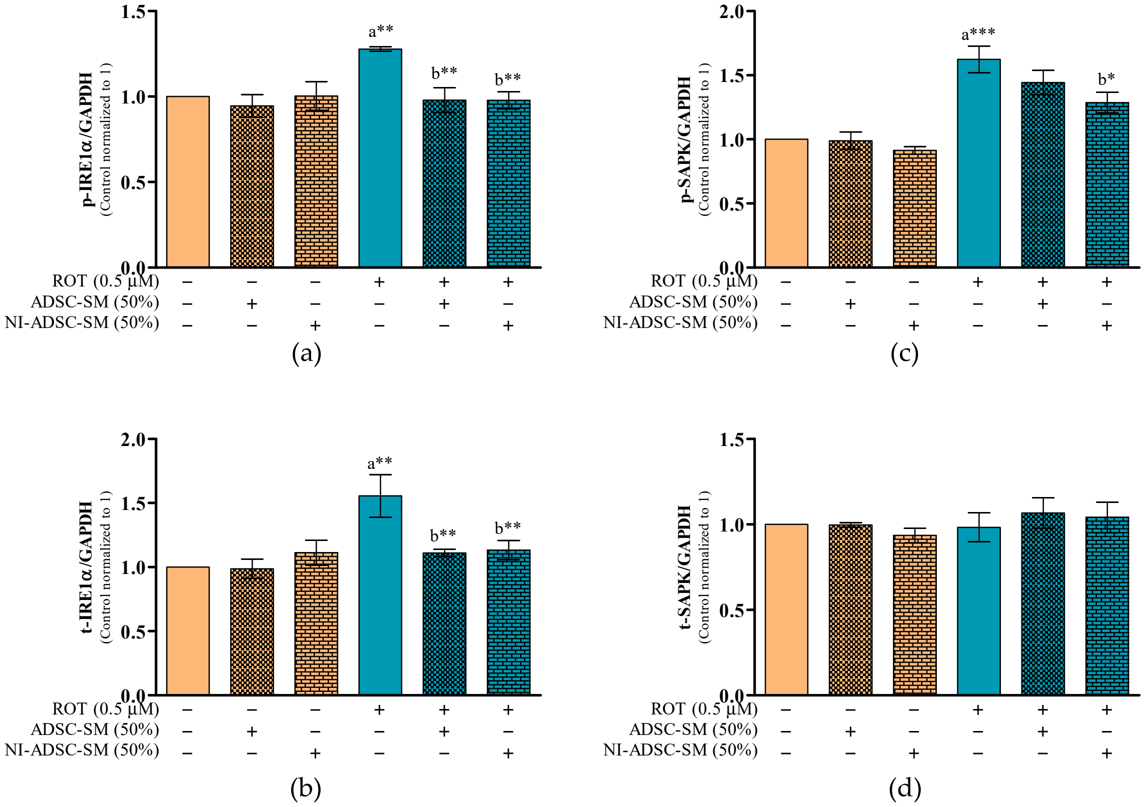

Supplementary Figure S4. SH-SY5Y cells were seeded as 50,000 cells/mL in DMEM containing 1% FBS and incubated overnight before experiments. Cells with presence of ROT (0.5 µM) or DMSO for 48 h were treated with ADSC-SM (50%) or NI-ADSC-SM (50%) during the last 24 h. The bar graphs represent the ratio for p-IRE1α Ser724/GAPDH (a), t-IRE1α/GAPDH (b), p-SAPK Thr183,Tyr185/GAPDH (c), and t-SAPK/GAPDH (d) by Western blotting. Data are shown as means (bars) and SEM (error bars) of three independent cell culture experiments, and were analyzed by one-way ANOVA with Tukey's post-test. Statistical comparison: a, compared with control; b, compared with ROT; * $p < 0.05$, ** $p < 0.01$, and *** $p < 0.001$.

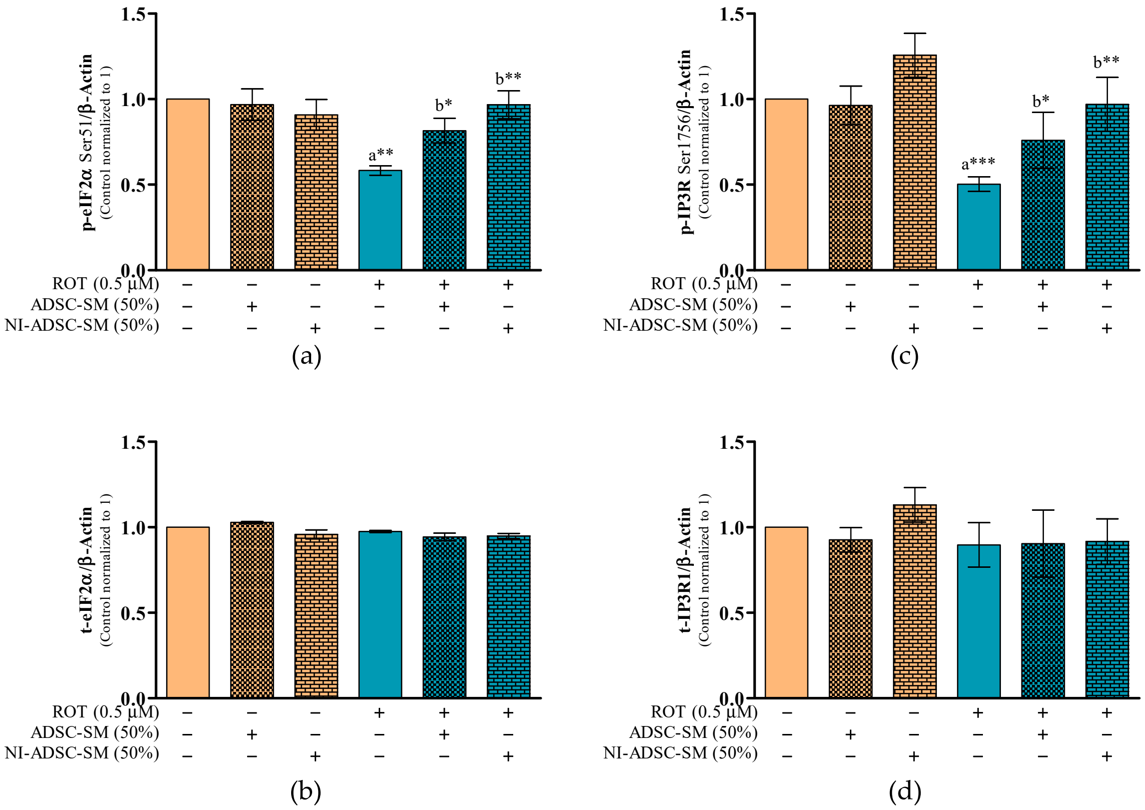

Supplementary Figure S5. SH-SY5Y cells were seeded as 50,000 cells/mL in DMEM containing 1% FBS and incubated overnight before experiments. Cells with presence of ROT (0.5 μM) or DMSO for 48 h were treated with ADSC-SM (50%) or NI-ADSC-SM (50%) during the last 24 h. The bar graphs represent the ratio for p-eIF2α/β-actin (a), t-eIF2α/β-actin (b), p-IP3R Ser1756/β-actin (c), and t-IP3R1/β-actin (d) by Western blotting. Data are shown as means (bars) and SEM (error bars) of three independent cell culture experiments, and were analyzed by one-way ANOVA with Tukey's post-test. Statistical comparison: a, compared with control; b, compared with ROT; * $p < 0.05$, ** $p < 0.01$, and *** $p < 0.001$.

Supplementary Table S1. Western blotting antibodies used in this study.

Antibody Name	Host, MW Details	Company	Cat. No.	Dilution
**Primary Antibodies:**				
LRRK2	Rabbit mAb, 290 kDa	Cell Signaling	#13046	1:1,000
PINK1	Mouse mAb, 66,52 kDa	Santa Cruz	sc-517353	1:500
Parkin	Mouse mAb, 50~58 kDa	Santa Cruz	sc-133167	1:300
Ubiquitin	Rabbit mAb, 9~300 kDa	Cell Signaling	#43124	1:1,000
DJ-1	Rabbit mAb, 22 kDa	Cell Signaling	#5933	1:3,000
TOM20	Rabbit mAb, 16 kDa	Cell Signaling	#42406	1:3,000
p-DRP1 (Ser616)	Rabbit mAb, 78~82 kDa	Cell Signaling	#4494	1:1,000
p-DRP1 (Ser637)	Rabbit pAb, 75~85 kDa	Biorbyt	orb127984	1:1,000
t-DRP1	Rabbit mAb, 78~82 kDa	Cell Signaling	#8570	1:1,000
MFN1	Mouse mAb, 86 kDa	Santa Cruz	sc-166644	1:500
MFN2	Mouse mAb, 86 kDa	Santa Cruz	sc-515647	1:500
OPA1	Rabbit pAb, 80~120 kDa	Abcam	ab90857	1:1,000
Bip (GRP78)	Rabbit pAb, 78 kDa	Cell Signaling	#3183	1:1,000
p-PERK (Thr980) HRP	Rabbit pAb HRP, 119 kDa	Biorbyt	orb504147	1:1,000
p-PERK (Thr981)	Rabbit pAb, 125 kDa	Biorbyt	orb336657	1:1,000
t-PERK	Rabbit mAb, 140 kDa	Cell Signaling	#3192	1:1,000
p-IRE-1α (Ser724)	Rabbit pAb, 130 kDa	Invitrogen	PA5-105424	1:1,000
t-IRE-1α	Rabbit mAb, 130 kDa	Cell Signaling	#3294	1:1,000
p-SAPK (Thr183/Tyr185)	Rabbit mAb, 46, 54 kDa	Cell Signaling	#4668	1:1,000
t-SAPK	Rabbit pAb, 46, 54 kDa	Cell Signaling	#9252	1:1,000
p-eIF2α (Ser51)	Rabbit mAb, 38 kDa	Cell Signaling	#3398	1:1,000
t-eIF2α	Rabbit mAb, 38 kDa	Cell Signaling	#5324	1:1,000
ATF4	Rabbit mAb, 49 kDa	Cell Signaling	#11815	1:1,000
CHOP	Mouse mAb, 27 kDa	Cell Signaling	#2895	1:1,000
p-IP3R (Ser1756)	Rabbit mAb, 320 kDa	Cell Signaling	#8548	1:1,000
t-IP3R1	Rabbit mAb, 320 kDa	Cell Signaling	#8568	1:1,000
GRP75	Rabbit pAb, 75 kDa	Cell Signaling	#2816	1:1,000
VDAC	Rabbit mAb, 32 kDa	Cell Signaling	#4661	1:1,000
GAPDH	Rabbit mAb, 37 kDa	Cell Signaling	#2118	1:3,000
GAPDH (HRP conjugate)	Rabbit mAb, 37 kDa	Cell Signaling	#8884	1:3,000
β-actin (HRP conjugate)	Rabbit mAB, 45 kDa	Cell Signaling	#5125	1:3,000
**Secondary Antibodies:**				
Anti-rabbit IgG, HRP-linked antibody		Cell Signaling	#7074	1:1,000
Anti-mouse IgG, HRP-linked antibody		Cell Signaling	#7076	1:1,000

p-, phosphorylated; t-, total.
pAb, polyclonal antibody; mAb, monoclonal antibody; kDa, kiloDalton.
